# Codon Adaptation of Plastid Genes

**DOI:** 10.1371/journal.pone.0154306

**Published:** 2016-05-19

**Authors:** Haruo Suzuki, Brian R. Morton

**Affiliations:** 1 Graduate School of Science and Engineering, Yamaguchi University, Yamaguchi, Japan; 2 Department of Biology, Barnard College, Columbia University, New York, New York, United States of America; Agriculture and Agri-Food Canada, CANADA

## Abstract

Codon adaptation is codon usage bias that results from selective pressure to increase the translation efficiency of a gene. Codon adaptation has been studied across a wide range of genomes and some early analyses of plastids have shown evidence for codon adaptation in a limited set of highly expressed plastid genes. Here we study codon usage bias across all fully sequenced plastid genomes which includes representatives of the Rhodophyta, Alveolata, Cryptophyta, Euglenozoa, Glaucocystophyceae, Rhizaria, Stramenopiles and numerous lineages within the Viridiplantae, including Chlorophyta and Embryophyta. We show evidence that codon adaptation occurs in all genomes except for two, *Theileria parva* and *Heicosporidium* sp., both of which have highly reduced gene contents and no photosynthesis genes. We also show evidence that selection for codon adaptation increases the representation of the same set of codons, which we refer to as the adaptive codons, across this wide range of taxa, which is probably due to common features descended from the initial endosymbiont. We use various measures to estimate the relative strength of selection in the different lineages and show that it appears to be fairly strong in certain Stramenopiles and Chlorophyta lineages but relatively weak in many members of the Rhodophyta, Euglenozoa and Embryophyta. Given these results we propose that codon adaptation in plastids is widespread and displays the same general features as adaptation in eubacterial genomes.

## Introduction

Codon usage bias, or the non-uniform representation of synonymous codons within a coding sequence, is a universal feature of genomes that arises from a combination of an underlying mutational bias and natural selection [1–3]. When considering codon usage bias a distinction must be made between the pattern, or ‘direction’, of codon bias–that is, the specific set of codons that are over-represented–and the degree of codon bias. Two genes can have the same degree of bias, measured as deviation from uniform representation of synonymous codons, but be biased towards a different set of codons and since mutation and selection can vary across a genome, genes within a genome can vary in both the degree and pattern of codon usage bias.

Mutation biases that shape genomic G+C content typically result in different genes within a genome displaying variation only in the degree of codon bias, not in the pattern of codon bias. In general, the underlying mutational process of a genome is biased either towards A+T or towards G+C and these two situations will lead to a pattern of codon usage bias in which the NNA and NNT codons (those with A or T at the third codon position) of all synonymous codon groups are over-represented or under-represented respectively. The degree to which this bias is observed in any individual gene within a genome can vary across the genome depending upon the variation in mutation bias across chromosome loci [4].

The contribution of natural selection to the codon usage of a gene can take two forms [5]. First, there can be selection at individual nucleotide sites that is independent of any protein-coding function of that site. We will consider this to be general background selection that can be folded into the mutational bias such that we can simply refer to the contribution of the substitution bias in a genome to codon usage. The second possible contribution of selection is dependent on the amino acid coding function of codons. In some genomes there is evidence that selection acts to increase the translation efficiency of certain genes by favoring a set of codons that are optimal for this process, with the implication that there are fitness differences between synonymous codons [2,6,7]. This action of selection is commonly thought to increase the representation of codons that yield the best trade-off between more rapid and more accurate translation by the available tRNA population in the cell [3,6,8,9], which could involve a co-evolution of codon usage and tRNA levels [10].

This second role for selection results in codon adaptation, which we define as an adaptation of the codon usage of a gene towards an increased representation of the codons that increase translation efficiency. These codons are referred to as adaptive codons. Codon adaptation has been observed in many organisms, particularly unicellular organisms [2,6]. The strength of this sort of selection varies amongst genes within a genome as a function of expression level with selection acting most strongly on highly expressed genes [2]. The result, in genomes where there is codon adaptation, is variation amongst genes in the pattern of codon usage as well as in the degree of bias towards the adaptive codons. In such genomes a large majority of genes show a codon usage pattern that can be largely attributed to the underlying substitution bias while a smaller number of highly expressed genes show a pattern of codon usage with an increased representation of adaptive codons. If codon adaptation and the substitution bias converge on the same codon usage pattern then adaptation can be more difficult to detect, but the two codon usage patterns are often distinguishable.

Given the influences of substitution bias and selection, or the general difference between degree and pattern of codon bias, we need to distinguish between codon adaptation and strict codon bias. Codon adaptation in the highly expressed, or highly translated, genes under strong selection will be manifest in a strong bias towards adaptive codons. If we consider a synonymous group with two codons, C_s_ and C_a_, where C_a_ is the adaptive codon, if the substitution bias is such that it would lead to C_s_ >> C_a_ then selection can result in C_a_ >> C_s_ in highly translated genes. However, genes under weaker selection can have levels of codon adaptation in which the action of selection somewhat offsets the action of the substitution bias such that C_a_ ≅ C_s_. In these cases there is codon adaptation since selection has led to an increased frequency of C_a_ but there is very little absolute codon bias. To account for the pattern of codon bias we can employ measures of codon usage, such as the Codon Adaptation Index (CAI, [11]), that measure the degree of bias towards a specified set of adaptive codons (such as C_a_, which must be determined separately, typically from codon usage in highly expressed genes) as opposed to statistics, such as the Effective Number of Codons (ENC, [12]), which measure only the degree of deviation from uniform codon usage regardless of which codons are over-represented. C_a_ >> C_s_ and C_s_ >> C_a_ both have high codon bias (low ENC) but the latter will have a low CAI. C_s_ >> C_a_ will have a higher codon bias (lower ENC) than C_a_ ≅ C_s_ but the latter will have a higher CAI.

In this study we investigate codon adaptation across a wide taxonomic range of sequenced plastid genomes. The plastid genome is a small genome encoding a limited set of genes that are fully expressed within the organelle. Evidence indicates that plastids are descended from a single cyanobacteria-like endosymbiont with the green plants, red algae and Glaucophytes retaining the descendant of this primary endosymbiont and at least two subsequent secondary endosymbiotic events giving rise to plastids in other lineages such as the Euglenozoa, Alveolata, Stramenopiles, Cryptophyta and Haptophyceae [13,14]. Among the 601 of the 605 completely sequenced and annotated plastid genomes at National Center for Biotechnology Information (NCBI, http://www.ncbi.nlm.nih.gov) as of September 2014, genome size ranged from 29,623 base pairs (bp) to 521,168 bp, with a median of 152,968 bp, and the protein-coding sequence (CDS) number ranged from 21 to 273 with a median of 84. (The other four of the 605 sequenced plastid genomes had no annotated CDS, which may indicate incomplete annotation and so they are excluded from this analysis.) Across different lineages there is a general substitution bias towards A and T with the sequenced genomes showing a %G+C content ranging from 13.7 to 57.7 with a median of 37.2; of the sequenced genomes only 3 have a %G+C greater than 50%. The general pattern of codon usage in plastid genes reflects this substitution bias with a high representation of NNA and NNT codons [15]. However, the substitution process in flowering plant chloroplasts is known to be strongly context-dependent in that the mutational dynamics of any given nucleotide are influenced by the composition of nucleotides flanking that site [16–18]. The result of this is that, despite a general bias towards NNA and NNT codons, the exact pattern of codon usage bias across synonymous codon groups is somewhat more complex in flowering plant chloroplasts [15,19]. Since it is not known if similar context effects exist in other plastids, the general bias towards NNA and NNT codons may mask similar complexity that we cannot consider in this study.

We perform an analysis of plastid gene codon usage in these plastid genomes with a focus on codon adaptation. Given that seed plant chloroplasts make up the vast majority of the sequenced genomes we limited their representation to a single dicot (*Nicotiana tabacum*), a single monocot (*Oryza sativa*) and a single gymnosperm (*Pinus thunbergii*) leaving a total of 103 genomes. We apply a uniform approach to all of the genomes and show evidence that highly translated genes, particularly *psbA* and *rbcL*, display codon adaptation in most plastid genomes and that selection favors the same, or a very similar, adaptive pattern of codon usage across all of the lineages. We also attempt to assess the strength of selection for codon adaptation, either in terms of the degree of bias towards the adaptive pattern or in the number of genes showing evidence for some degree of codon adaptation, across the plastid genomes. In general we find that Rhodophyta, with the exception of the Bangiales, and seed plants (represented in our study by *Nicotiana tabacum*, *Oryza sativa*, and *Pinus thunbergii*) have relatively low, but detectable, levels of codon adaptation while Stramenopiles and Chlorophyta in particular have relatively strong codon adaptation.

The results lead us to propose that all plastid codon usage can be largely explained by a single general model of substitution bias towards A+T and codon adaptation to the same set of codons in all plastids. This adaptation is based on the limited plastid tRNA population. The degree of adaptation towards these favored codons varies across genomes and across genes as a function of some aspect of gene expression, most likely translation efficiency. Although there are likely to be additional factors that make minor contributions to the variation in codon usage amongst genes within any specific genome, our data indicate that codon adaptation is a common feature of plastid genomes.

## Materials and Methods

### Software and Databases

Most analyses described below were implemented on the G-language Genome Analysis Environment version 1.9.0 [20–22], available at http://www.g-language.org. The exception is the codon resampling test, which was performed using a Java Package written by BRM. Statistical computing and graph drawing were conducted with R version 3.1.2 [23], available at http://www.R-project.org.

A list of the 103 plastid genomes along with taxanomic information is given in S1 Table. All genome sequences were taken from NCBI FTP Site (ftp://ftp.ncbi.nlm.nih.gov/genomes/) in September 2014. tRNA genes encoded in the 38 plastid genomes were retrieved from the tRNA Gene DataBase Curated by Experts "tRNADB-CE" [24], available at http://trna.ie.niigata-u.ac.jp/.

### Codon fitness values

Codon fitness values, also called the relative adaptiveness of codons [11], are typically calculated from the codon usage of highly expressed genes. Based on the fact that product of the *psbA* gene is the major translation product in chloroplast [25] we used the *psbA* gene from a small set of taxa to estimate codon fitness. For each codon, fitness is calculated by dividing the usage of that codon by the maximum usage within the synonymous group. A codon with no representation in the reference group is assigned a fitness value of 0.002 as in [11]. For any gene, or cumulative set of genes, the geometric mean of the codon fitness values of the codon usage table is the Codon Adaptation Index (CAI). The use of high expression genes to define fitness values results in a CAI that is a measure of adaptation for expression, more specifically translation efficiency [11] but more generally, CAI as an information statistic can be used to measure the degree of fit to any defined codon usage pattern. The codon fitness values used in this study are listed in S2 Table. The number of high expression genes used to estimate codon fitness values was limited so that the CAI value of a gene would not generally utilize codon fitness values derived from that same gene or from genes from the same genome. However, altering the set of high expression genes used to infer codon fitness values did not substantively alter any of the results (data not shown).

### Codon resampling test

We applied a resampling method to each genome to test for genes with codon adaptation levels significantly above what would be generated by the genome substitution bias. The null hypothesis is that all genes within the genome have the same level of codon adaptation; this would be the level of adaptation arising from the genome substitution bias. Therefore, if variation in substitution bias is minimal across the small genome, then the codon usage in each gene would represent a random sample from the same codon distribution. To test this hypothesis the codons from every protein-coding sequence from a given genome were pooled. For every gene we generated a random codon usage by drawing with replacement from this pool until the sampled codons had the same amino acid usage as the gene and calculated CAI for this random codon usage using the codon fitness values described above. This resampling was repeated 1000 times for each gene to yield an expected distribution of CAI for that gene under the null hypothesis. A gene was rejected if the observed CAI was more than 3 standard deviations greater than expected.

Once we had run the resampling on every gene within a genome we repeated the test leaving out all genes rejected in the previous round. This was repeated until no genes were rejected in a round indicating that the level of codon adaptation in each of the remaining genes could be explained by the same codon usage. All genes rejected in this resampling were considered to have evidence for significant codon adaptation. The protocol was performed using a Java Package written by BRM.

### Hierarchical clustering of genes based on codon usage

Dendrograms were constructed using hierarchical clustering (Unweighted Pair Group Method with Arithmetic mean; UPGMA) based on dissimilarity in codon usage between genes. Dissimilarity between two genes based on 59 variables of codon usage was measured using Pearson correlation distance (one minus Pearson product-moment correlation coefficient). Absolute codon frequencies (codon count data) were used for clustering genes putatively translated at high (*psbA*, *rbcL*, and *psbC*) and low (*rps3*, *rps4*, and *rpoB*) levels from 43 plastid genomes. To control for amino acid composition, codon usage data were normalized by dividing the usage of each codon by the maximum usage in each amino acid. The normalized codon usage data were used in the analysis of all *psbA* and *psbN* genes.

### The strength of selection for codon adaptation (S)

Following [7] and [26], an S value was calculated for each plastid genome using the codon frequencies for four amino acids, Phe (C_a_ = TTC and C_s_ = TTT), Tyr (C_a_ = TAC and C_s_ = TAT), Ile (C_a_ = ATC and C_s_ = ATT), and Asn (C_a_ = AAC and C_s_ = AAT), where the two codons (C_s_ and C_a_) are recognized by the same tRNA species but C_a_ is recognized more efficiently. The S is based on a comparison of codon frequencies within these synonymous groups between high expression genes and all other genes ([7] and [26]). Given the small number of genes coded by the plastid genome we used only *psbA* as the reference highly expressed gene to calculate S values for all 103 plastids. A second analysis used three genes, *psbA*, *rbcL* and *psbC* as the reference highly expressed genes and the value generated in this case referred to as S_3_.

### Within-group correspondence analysis (WCA) of codon usage

WCA combines multivariate data into a small number of variables (axes) that explains most of the variation among the original variables [27,28]. In our study our variables are the 18 codons for 9 two-fold degenerate amino acids (C, D, E, F, H, K, N, Q, and Y by the single letter amino acid code) for each gene in a genome, and WCA yields the coordinates of each gene on each new axis.

### Replication strand skew analysis

The degree of replication strand bias was measured by the GC skew index (GCSI) [29,30]. The GCSI was calculated with a window number of 256, considering that each window should contain at least 100 bp and that the genome sizes for the plastid genomes ranged from 29,623 to 521,168 bp. The GCSI can take values from 0 (no bias) to approximately 1 (high bias), and empirically a genome with a clear GC skew has a GCSI of > 0.1. For the plastid genomes with a GCSI over 0.1, the origin and terminus of DNA replication were predicted using cumulative GC skew [31] so that genes were located on the leading or lagging strand.

## Results

### Codon usage patterns in plastid genes

Previous analyses of a limited number of plastid genes showed evidence for two main patterns of codon usage, one of which was proposed to be a result of codon adaptation [15,25]. The two codon usage patterns are illustrated here using the codon usage tables from three widely separated taxa, the liverwort *Marchantia polymorpha*, the green alga *Chlamydomonas reinhardtii* and the red alga *Porphyra purpurea* (Table 1). One codon usage pattern is observed in the cumulative codon usage of each genome. This pattern is a general bias towards the NNA and/or NNT codons within each synonymous group and is consistent with the general bias across plastid genomes [15,19], likely due to a general substitution bias.

**Table 1:** Codon usage in three plastid genomes.

Codon^1^	tRNA^2^	Mpo *psbA*	Mpo Total	Cre *psbA*	Cre Total	Ppu *psbA*	Ppu Total
AGT	0	3	405	0	306	5	772
**AGC**	38	**7**	**47**	**2**	**91**	**7**	**333**
AAT	0	7	1219	1	792	2	1925
**AAC**	38	**14**	**171**	**24**	**410**	**19**	**440**
TAT	0	2	802	0	495	5	1093
**TAC**	38	**11**	**93**	**13**	**226**	**8**	**312**
TTT	0	8	1518	2	638	8	1445
**TTC**	38	**17**	**99**	**24**	**406**	**17**	**437**
CAT	0	5	379	1	197	3	615
**CAC**	38	**5**	**62**	**9**	**220**	**7**	**175**
ATT	0	17	1480	5	1129	13	2407
**ATC**	36	**12**	**98**	**23**	**199**	**12**	**453**
ATA	0	0	695	0	111	0	1101
TGT	0	0	207	3	167	0	299
**TGC**	36	**2**	**38**	**1**	**17**	**2**	**143**
GAT	0	4	709	1	514	5	1684
**GAC**	38	**4**	**72**	**6**	**185**	**2**	**365**
ACA	38	1	477	4	656	8	1027
ACT	0	14	597	12	534	8	1081
ACC	23	2	58	0	59	1	183
ACG	0	0	41	0	72	0	153
CCA	38	3	355	8	467	11	701
CCT	0	12	459	4	323	5	757
CCC	4	0	38	0	34	0	89
CCG	0	0	47	2	51	0	130
GCA	38	6	438	7	460	10	1242
GCT	0	32	752	25	812	26	1432
GCC	3	0	62	0	78	1	249
GCG	0	0	47	0	65	0	182
GGA	33	3	658	0	160	6	1108
GGT	0	29	612	30	1076	23	1233
GGC	35	1	82	1	97	3	447
GGG	0	0	88	0	68	0	225
GTA	38	13	442	16	599	14	1020
GTT	0	11	627	5	615	13	1391
GTC	21	0	47	0	12	0	250
GTG	0	0	46	0	74	0	225
TCA	38	4	350	16	454	1	679
TCT	0	12	614	12	392	15	1176
TCC	23	0	71	0	37	3	196
TCG	7	0	48	0	74	0	98
CTA	38	2	141	6	141	18	818
CTT	0	8	507	8	319	3	700
CTC	11	0	24	0	9	0	126
CTG	0	0	25	0	40	0	212
TTA	37	15	1823	18	1617	12	2255
TTG	30	5	199	0	75	2	475
CGA	0	0	248	0	67	0	285
CGT	38	10	344	15	734	11	330
CGC	0	2	46	0	57	2	123
CGG	23	0	22	0	4	0	56
AGA	38	2	367	0	99	1	1152
AGG	4	0	24	0	15	0	181
GAA	37	17	1080	15	914	16	2133
GAG	0	2	84	4	76	2	490
AAA	37	0	1724	0	1497	1	2417
AAG	0	1	81	0	86	0	582
CAA	38	6	855	7	782	7	1475
CAG	0	0	51	0	63	2	401

1 –Codon usage is given for the *psbA* gene and all genes cumulatively (total) for *Marchantia polymorpha* (Mpo), *Chlamydomonas reinhardtii* (Cre) and *Porphyra purpurea* (Ppu). The NNC codons of the two-fold degenerate groups are in bold: the *psbA* genes have an increased frequency of these codons as discussed in the text. The AGT and AGC codons of Serine are grouped with the NNY two-fold degenerate codon groups separate from the TCN Serine codons.

2 –Number of the 38 plastid genomes in the tRNA database at http://trna.ie.niigata-u.ac.jp/ that have a tRNA complementary to the codon.

The highly expressed *psbA* gene displays a second general codon usage pattern as observed in Table 1. Although the *psbA* gene, which codes a highly translated core photosystem II protein, differs from the cumulative codon usage pattern of its genome, the codon usage patterns of the three *psbA* genes are similar to one another. The most obvious difference between the two codon usage patterns–that is, between the cumulative codon usages and the codon usage of the *psbA* genes—is in the two-fold degenerate NNY codon groups (i.e. two-fold degenerate codon groups with a third position pyrimidine). In these synonymous groups we see a relatively high frequency of the NNC codons in *psbA* when compared to the cumulative bias towards NNT (Table 1). Although this bias towards NNC is particularly obvious there are other differences between the two codon usage patterns; for example, unlike in the cumulative codon usage pattern the *psbA* genes do not utilize either CGG or CGA for Arginine, nor do they use GGG to code glycine, and there is a strong bias towards coding glycine with GGT instead of GGA.

**Fig 1:**
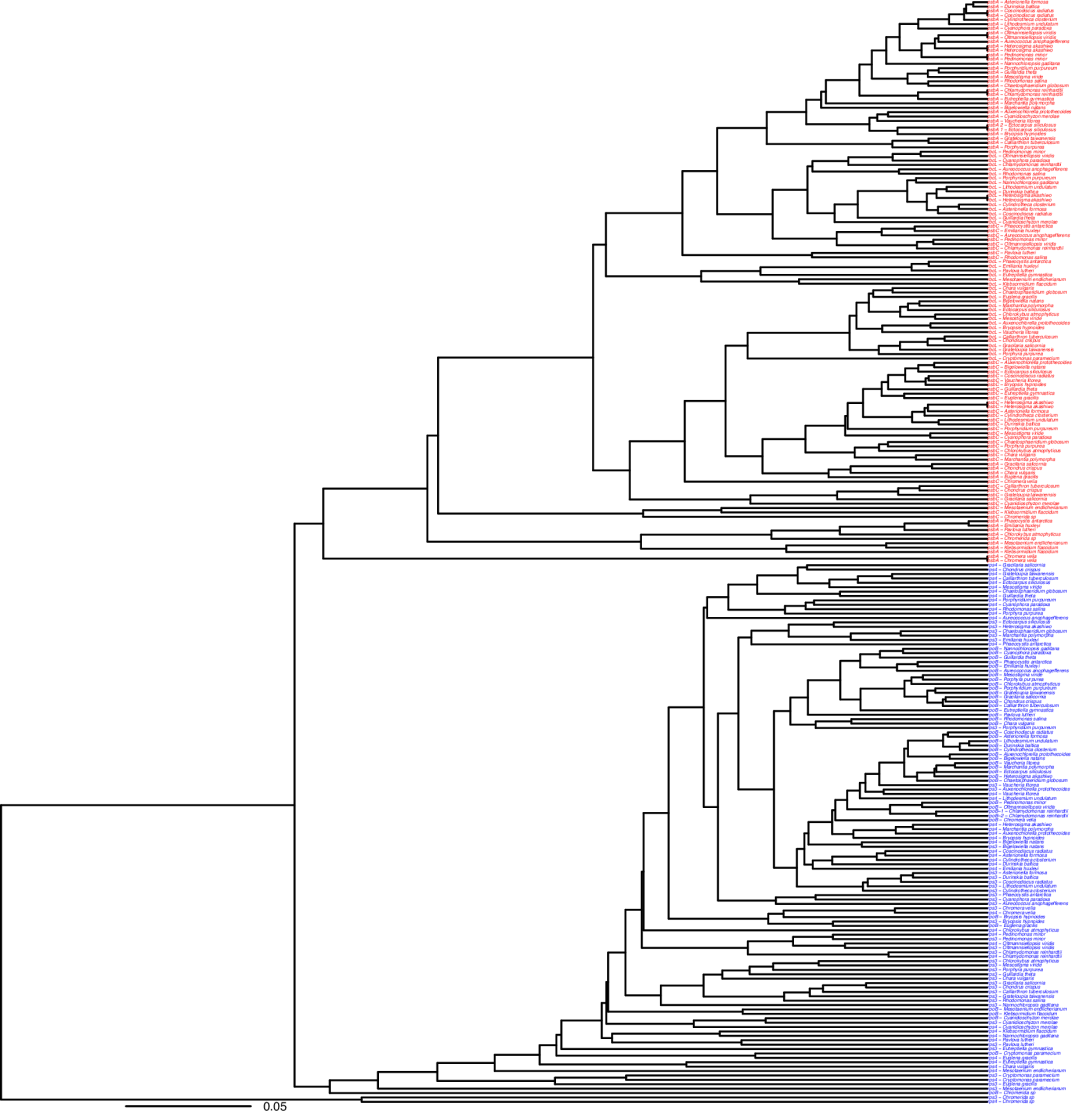
Codon usage patterns in plastid genes. A cluster of three putative high-translation (*psbA*, *rbcL*, *psbC* in red) and three putative low-translation (*rps3*, *rps4*, *rpoB* in blue) genes from 43 plastid genomes selected to represent the major lineages (see text). Genes are clustered by similarity in codon usage as described in the Materials and Methods.

To examine these two codon usage patterns across a broader spectrum of taxa, we constructed a dendrogram by clustering a set of high and low expression genes based on similarity of codon usage. The former set was composed of two highly translated genes, *psbA*, and *rbcL*, which codes the large subunit of RuBisCO [15,19,25], and a third gene, *psbC*, that codes a core component of photosystem II and so is likely to have a relatively high level of translation. For comparison to these we chose three plastid genes expected to have relatively low translation levels [15]. We clustered these genes from each of 43 genomes (Fig 1) that were randomly selected with the goal of having a representative of each Order while reducing the density of the cluster at the same time.

**Fig 2:**
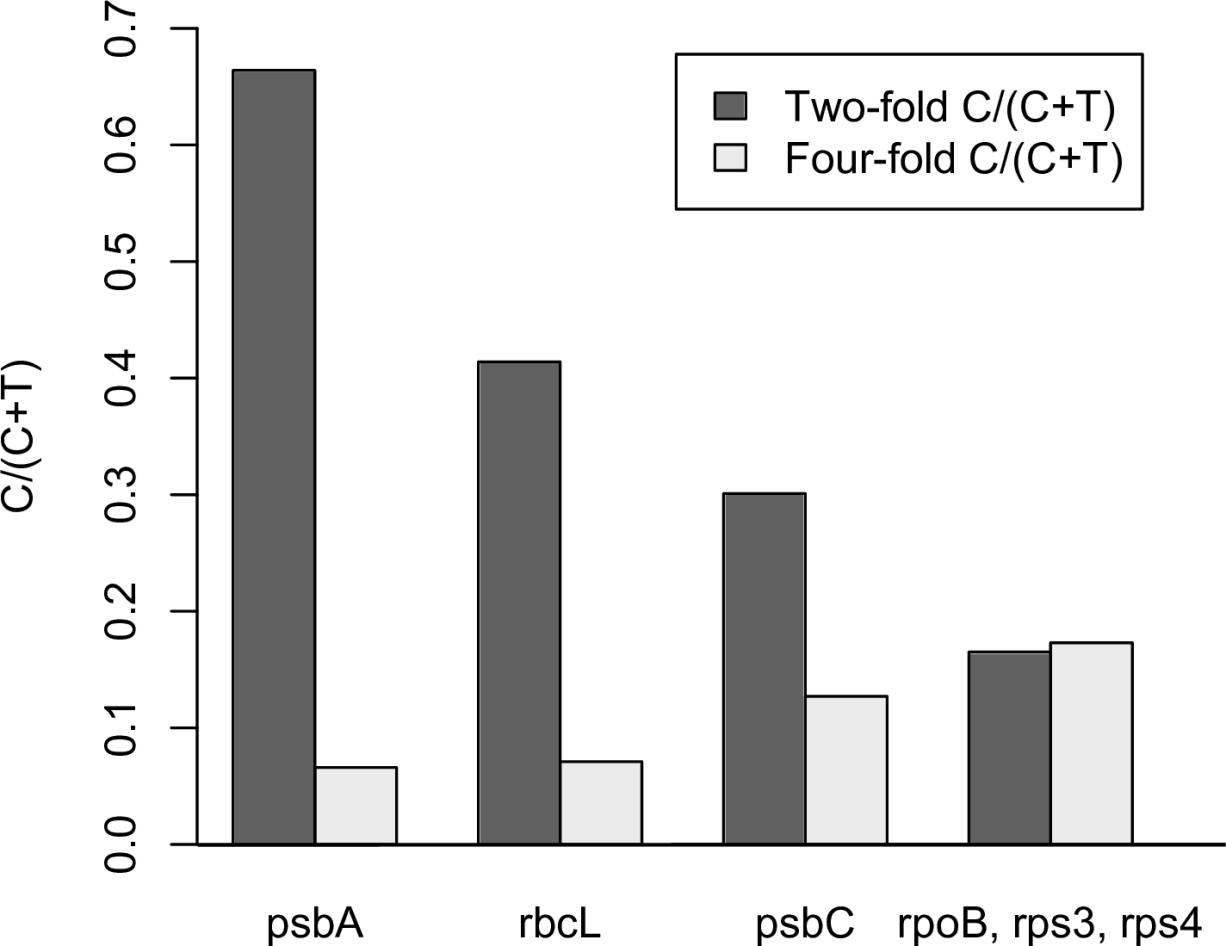
Third position composition patterns. A plot of %C (C/[C+T]) base composition at two-fold degenerate and four-fold degenerate sites for the genes shown in Fig 1. Values are the cumulative base composition for each gene. For the low translation genes we show the cumulative composition of the *rps3*, *rps4* and *rpoB* genes.

The codon usages of the genes in Fig 1 show that the pattern of codon usage in the *psbA* genes in Table 1 is observed in high expression genes across a wide taxanomic range. Two major clusters of genes are apparent in Fig 1, one composed of the high translation genes and the other composed of the low translation genes. The differences in codon usage pattern between the low and high translation genes are the same as the differences between the codon usage patterns given in Table 1 (S3 Table). For simplification these differences are reduced to two general compositional differences, the increased frequency of NNC codons within twofold degenerate groups and the higher frequency of NNT codons in fourfold degenerate codon groups, and presented in Fig 2. As with the differences observed in Table 1, the *psbA* genes have a total of 66.4% NNC within the two-fold degenerate NNY groups while the low translation genes have a total of just 16.5% NNC in these groups. This increased frequency of NNC in the NNY codon groups is also apparent to a lesser degree in the high translation *rbcL* (41.4% NNC) and *psbC* (30.1% NNC) genes. In addition, this bias towards NNC in the genes within the high expression genes does not extend to the four-fold degenerate codon groups. Instead there is a lower frequency of NNC in these codon groups relative to the other genes (Fig 2). Consistent with previous proposals [15,25] we interpret this as evidence of codon adaptation in highly expressed genes across a wide range of taxa. This proposal will be discussed in detail below.

### Variation in levels of codon adaptation across plastid genomes

If the general codon usage pattern in the *psbA* genes in Table 1 and S3 Table is an adaptive pattern and selection favors the same adaptive pattern of codon usage across plastid lineages, then we can try estimate the relative strength of selection acting on different genomes by measuring the degree to which highly expressed genes are biased towards the adaptive pattern. The strength of selection could be manifest in the degree to which selection is increasing the codon adaptation of high expression/translation genes within a genome and/or in the number of genes that are under some level of selective pressure to increase the representation of adaptive codons above the level that would result from the substitution bias alone. We examined this using three different metrics that can measure one or the other, or both, of these two aspects of codon adaptation; a codon resampling test [15], the calculation of a genome selection coefficient, S, following [7] and comparison of CAI values based on the single set of codon fitness values as described in the Materials and Methods. Based on the three metrics we then put forward a single statistic, called S_pca_, to estimate relative selection strength within a genome. The full results of the analyses are given in S1 Table and the results of the three approaches are discussed separately below.

#### Codon resampling test

The resampling test results are summarized in Tables 2 and 3. In this test, resampling with replacement from a combined genome pool of codons from all identified protein-coding sequences was used to estimate an expected level of codon adaptation for individual genes in that genome. As discussed in the Materials and Methods, rejection indicates that the level of adaptation is significantly higher than expected were the gene composed of codons representative of the genome-wide codon pool. The resampling test showed a strong trend towards rejecting highly expressed genes (Table 2) and there is striking similarity across genomes in what genes display a significantly increased CAI values, or codon adaptation. The *psbA* gene, the major translation product in plant chloroplasts, was rejected in the resampling test of every genome except *Cyanidium caldarium* [32], one of the few genomes in which *rbcL* was also not rejected. Other genes that were rejected in a majority of genomes were the small subunit of RuBisCo (*rbcS*), *tufA*, which codes a translation elongation factor [33], the *cpeA* and *cpeB* genes coding for the two subunits of the Phycoerythrin protein involved in light harvesting (see http://www.ncbi.nlm.nih.gov/gene/810008 and http://www.ncbi.nlm.nih.gov/gene/856988 respectively), *psbC* (discussed above), and another major gene for photosystem II *psbD* [34].

**Table 2:** Genes with highest rejection rate across genomes in the resampling analysis.

Gene	Number of Genomes^1^	Number of Genomes Rejected^2^
*psbA*	98	97 (99.0%)
*rbcL*	98	86 (87.8%)
*cpeB*	12	10 (83.3%)
*cpeA*	10	8 (80.0%)
*rbcS*	48	34 (70.83%)
*tufA*	92	62 (67.4%)
*psbC*	96	61 (63.5%)
*psbD*	97	58 (59.8%)

1 –Number of genomes that code the gene.

2 –Number of genomes in which the gene was rejected in the resampling analysis.

**Table 3:** Genomes with the highest levels of rejection in the resampling analysis.

Genome	Classification	Number of Genes Rejected	Genes^1^
**Highest 10**			
*Gonium pectorale*	Chlorophyta, Chlorophyceae	50 (74.6%)	
*Chlamydomonas reinhardtii*	Chlorophyta, Chlorophyceae	45 (61.2%)	
*Dunaliella salina*	Chlorophyta, Chlorophyceae	37 (53.0%)	
*Pleodorina starrii*	Chlorophyta, Chlorophyceae	37 (47.8%)	
*Oltmannsiellopsis viridis*	Chlorophyta, Oltmannsiellopsis	41 (47.5%)	
*Monomastix sp*.	Chlorophyta, Prasinophytes	64 (43.8%)	
*Klebsormidium flaccidum*	Streptophyta, Klebsormidiophyceae	44 (43.3%)	
*Schizomeris leibleinii*	Chlorophyta, Chlorophyceae	29 (37.3%)	
*Stigeoclonium helveticum*	Chlorophyta, Chlorophyceae	29 (37.3%)	
*Pedinomonas minor*	Chlorophyta, Pedinophyceae	28 (34.2%)	
**Lowest 10**			
*Cryptomonas paramecium*	Cryptophyta, Cryptomonadales	2 (2.6%)	*rbcL rpl3*
*Calliarthron tuberculosum*	Rhodophyta, Florideophyceae	5 (2.6%)	*psbA rbcL cpeB cpeA psb30*
*Grateloupia taiwanensis*	Rhodophyta, Florideophyceae	4 (2.1%)	*psbA*, *rbcL*, *cpeA*, *cpeB*
*Gracilaria salicornia*	Rhodophyta, Florideophyceae	4 (1.9%)	*psbA*, *rbcL*, *cpeA*, *psbE*
*Pinus thunbergii*	Streptophyta, Embryophyta	3 (1.9%)	*psbA*, *rbcL*, *psbD*
*Gracilaria tenuistipitata*	Rhodophyta, Florideophyceae	4 (1.6%)	*psbA*, *rbcL*, *apcB*, *atpA*
*Cyanidium caldarium*	Rhodophyta, Bangiophyceae	3 (1.5%)	*rpl19*, *rps5*, *rpl24*
*Galdieria sulphuraria*	Rhodophyta, Bangiophyceae	2 (1.1%)	*psbA*, *cemA*
*Theileria parva*	Alveolata, Apicomplexa	0	N/A
*Helicosporidium sp*.	Chlorophyta, Trebouxiophyceae	0	N/A

1 –For genomes with the lowest rejection rates those genes rejected are listed

Of the genes coded by at least 85 of the genomes we found the lowest rejection rates for *psbN* (1 of 97 genomes) a gene of unknown function associated with the biogenesis of both photosystems [35], *rpoC2* (2 of 97 genomes) a component of the plastid RNA polymerase [36], *ycf4* (2 of 93 genomes), which codes a non-essential assembly factor of photosystem I [37], *rpl20* and *rpl36* (3 of 98 each), both encoding ribosomal proteins, *rpoA* (3 of 95) and *rpoC1* (3 of 91), both of which encode plastid RNA polymerase components [38], *ccsA* (3 of 86), which appears to encode a protein involved in heme attachment to chloroplast cytochromes [39], *rpl23* (4 of 95), encoding a ribosomal protein and *ycf3* (4 of 87), whose function is unknown but may be related to photosystem biogenesis [37].

Table 3 shows the number of genes rejected within each genome. The plastid genomes with the highest levels of rejection (i.e. fraction of genes rejected) are all from Chlorophytes, and the four plastid genomes with the highest rejection levels, *Gonium*, *Chlamydomonas*, *Dunaliella* and *Pleodorina*, are all from members of the Chlamydomonadales. These data indicate that within some lineages, particularly some of the green algae, a large number of plastid genes are under selection for codon adaptation. The plastid genomes with the lowest rejection rates are predominantly from Rhodophytes with notable exceptions being pine (*Pinus thunbergii*) and the non-photosynthetic *Cryptomonas paramecium*, which does not code a *psbA* gene. For the genomes with the lowest rejection rates the results indicate that essentially all genes are equivalent in terms of codon usage and it is most likely that their codon usage is determined almost exclusively by substitution bias. The few genes that are rejected in these genomes are genes such as *psbA*, *rbcL*, *cpeA* and *cpeB* that are widely rejected, and the rejection is indicative that they differ significantly from the cumulative codon pool. Two genomes, one from the bovine pathogen *Theileria parva* [40] and the other from the Chlorophyte *Helicosporidium* sp. [41], showed no evidence for significant codon adaptation in any gene. *Theileria* has a highly reduced genome of just 39,579 nucleotides and does not code any photosystem I or photosystem II gene, nor does it code *rbcL*. *Helicosporidium* is a parasitic green alga that also has a highly reduced genome of just 37,454 nucleotides. Like *Theileria* it does not code any photosystem genes or *rbcL*.

#### Genome S values

The results of the S calculations are summarized in Table 4. S provides information on the strength of selection on those genes under selection as measured by the degree to which they differ from the overall genome codon usage. This is in contrast to the resampling which is more informative about the breadth, in terms of the number of genes affected, of selection.

**Table 4:** Genomes with the Lowest and Highest S Coefficients.

Genome	Classification	S
**Highest 15**		
*Chaetosphaeridium globosum*	Streptophyta, Coleochaetophyceae	3.405
*Coscinodiscus radiatus*	Stramenopiles, Bacillariophyta	3.146
*Vaucheria litorea*	Stramenopiles, PX_clade	2.998
*Mesostigma viride*	Streptophyta, Mesostigmatophyceae	2.989
*Marchantia polymorpha*	Streptophyta, Embryophyta	2.973
*Pseudendoclonium akinetum*	Chlorophyta, Ulvophyceae	2.953
*Phaeodactylum tricornutum*	Stramenopiles, Bacillariophyta	2.752
*Asterionella formosa*	Stramenopiles, Bacillariophyta	2.745
*Schizomeris leibleinii*	Chlorophyta, Chlorophyceae	2.743
*Roya anglica*	Streptophyta, Zygnemophyceae	2.640
*Fucus vesiculosus*	Stramenopiles, PX_clade	2.604
*Porphyra yezoensis*	Rhodophyta, Bangiophyceae	2.568
*Chlorella sorokiniana*	Chlorophyta, Trebouxiophyceae	2.564
*Guillardia theta*	Cryptophyta, Pyrenomonadales	2.545
*Pyropia haitanensis*	Rhodophyta, Bangiophyceae	2.452
**Lowest 15**		
*Grateloupia taiwanensis*	Rhodophyta, Florideophyceae,	1.621
*Cyanidioschyzon merolae*	Rhodophyta, Bangiophyceae,	1.554
*Monomorphina aenigmatica*	Euglenozoa, Euglenida,	1.501
*Pinus thunbergii*	Streptophyta, Embryophyta	1.414
*Nicotiana tabacum*	Streptophyta, Embryophyta	1.226
*Chondrus crispus*	Rhodophyta, Florideophyceae	1.204
*Euglenaformis proxima*	Euglenozoa, Euglenida	1.062
*Oryza sativa*	Streptophyta, Embryophyta	1.026
*Chromera velia*	Alveolata, Chromerida,	1.010
*Gracilaria tenuistipitata*	Rhodophyta, Florideophyceae	0.843
*Chara vulgaris*	Streptophyta, Charophyceae	0.816
*Gracilaria salicornia*	Rhodophyta, Florideophyceae	0.814
*Galdieria sulphuraria*	Rhodophyta, Bangiophyceae	0.566
*Cyanidium caldarium*	Rhodophyta, Bangiophyceae	0.562
*Euglena gracilis*	Euglenozoa, Euglenida	0.453

Overall, the genomes with low S values are from Rhodophyta, including *Cyanidium*, which was the only genome that did not reject either *psbA* or *rbcL* in the resampling analysis, Euglenozoa and Embryophyta. Taxa with high S values are from a number of orders and include representatives of the Stramenopiles, Chlorophyta and basal lineages within the Streptophyta. Interestingly, the Rhodophyta have representatives with both low and high S values. The members of the Bangiales, represented here by *Porphyra* and *Pyropia*, have high S values while the other representatives of the Bangiophyceae—*Cyanidioschyzon*, *Cyanidium* and *Galderia* which are Cyanidiales, and *Porphyridium* which is a member of the Porphyridiales–as well as the members of the Florideophyceae have low S values.

One difficulty with using just the *psbA* gene to calculate S is that, although it is the best gene to use in terms of codon adaptation, the amino acid composition pattern of this gene results in an invalid S in several genomes. Therefore, we repeated the analysis using *psbA*, *rbcL* and *psbC* as the high translation genes (Table 5) to generate a second S value that we call S_3_. The genomes with the lowest S_3_ values are essentially the same as those with the lowest S values but the genomes with the highest S_3_ values are different. Since none of the 15 genomes with the highest S_3_ values had a valid S and some of the genomes with the highest S values did not have a valid S_3_ (S1 Table) the two sets of putative strong selection genomes are not directly comparable but we interpret either a high S or a high S_3_ as an indication of strong selection.

**Table 5:** Genomes with the Lowest and Highest S_3_ Coefficients^1^.

Genome	Classification	S_3_
**Highest 15**		
*Asterionellopsis glacialis*	Stramenopiles, Bacillariophyta	3.093
*Kryptoperidinium foliaceum*	Alveolata, Dinophyceae	2.434
*Dunaliella salina*	Chlorophyta, Chlorophyceae	2.418
*Emiliania huxleyi*	Haptophyceae, Isochrysidales	2.385
*Prasinoderma coloniale*	Chlorophyta, Prasinophytes	2.384
*Leptocylindrus danicus*	Stramenopiles, Bacillariophyta	2.332
*Trebouxiophyceae sp*.	Chlorophyta, Trebouxiophyceae	2.330
*Odontella sinensis*	Stramenopiles, Bacillariophyta	2.200
*Aureococcus anophagefferens*	Stramenopiles, Pelagophyceae	2.181
*Monomastix sp*.	Chlorophyta, Prasinophytes	2.164
*Durinskia baltica*	Alveolata, Dinophyceae	2.127
*Lithodesmium undulatum*	Stramenopiles, Bacillariophyta	2.097
*Didymosphenia geminata*	Stramenopiles, Bacillariophyta	2.082
*Phaeocystis globosa*	Haptophyceae, Phaeocystales	1.965
*Pycnococcus provasolii*	Chlorophyta, Prasinophytes	1.939
**Lowest 15**		
*Grateloupia taiwanensis*	Rhodophyta, Florideophyceae	1.046
*Calliarthron tuberculosum*	Rhodophyta, Florideophyceae	1.040
*Chondrus crispus*	Rhodophyta, Florideophyceae	1.015
*Mesotaenium endlicherianum*	Streptophyta, Zygnemophyceae	0.914
*Monomorphina aenigmatica*	Euglenozoa, Euglenida,	0.838
*Nicotiana tabacum*	Streptophyta, Embryophyta	0.730
*Euglenaformis proxima*	Euglenozoa, Euglenida	0.676
*Pinus thunbergii*	Streptophyta, Embryophyta	0.667
*Gracilaria salicornia*	Rhodophyta, Florideophyceae	0.626
*Cyanidium caldarium*	Rhodophyta, Bangiophyceae	0.624
*Gracilaria tenuistipitata*	Rhodophyta, Florideophyceae	0.576
*Oryza sativa*	Streptophyta, Embryophyta	0.474
*Euglena gracilis*	Euglenozoa, Euglenida	0.395
*Galdieria sulphuraria*	Rhodophyta, Bangiophyceae	0.269
*Chara vulgaris*	Streptophyta, Charophyceae	0.178

1 –The S_3_ coefficient as defined in the text.

#### Maximal Codon Adaptation Index (CAI)

Selection strength can also be assessed from the maximum CAI value observed for a gene in any given genome (Table 6), which is *psbA* in almost every genome (data not shown). As with the S statistic, the maximum CAI value provides information about the strength of selection on highly expressed genes but not about the number of genes that are under selection. Since all CAI values are based on the single set of codon fitness values (see Materials and Methods), they are comparable among different genes and genomes. Taxa with the highest maximal CAI values are predominantly members of the Chlorophyta, Stramenopiles and Alveolata. Genomes with low maximal CAI values are predominantly vascular plants and members of the Rhodophyta meaning that codon adaptation is relatively weak in these taxa. One interesting case is the Chlorophyte *Trebouxiophyceae sp*. which has a low maximal CAI. This genome also had a low rejection rate in the resampling analysis but it ranked relatively high in the S_3_ analysis. Of interest is that this is the only plastid genome in the analysis with a genome GC content over 50% (57.5%) and the only one with a GC3 content over 50% (63.5%). Since the calculation of S is based on the difference between genome composition and the composition of designated genes, the S_3_ result for this species is probably a result of its unusual genome composition.

**Table 6:** Genomes ranked by the maximal CAI value.

Genome	Classification	Max. CAI
**Highest 15**		
*Oltmannsiellopsis viridis*	Chlorophyta, Oltmannsiellopsis	0.915
*Asterionellopsis glacialis*	Stramenopiles, Bacillariophyta	0.877
*Kryptoperidinium foliaceum*	Alveolata, Dinophyceae	0.859
*Durinskia baltica*	Alveolata, Dinophyceae	0.856
*Leptocylindrus danicus*	Stramenopiles, Bacillariophyta	0.844
*Odontella sinensis*	Stramenopiles, Bacillariophyta	0.831
*Pedinomonas minor*	Chlorophyta, Pedinophyceae	0.830
*Thalassiosira oceanica*	Stramenopiles, Bacillariophyta	0.828
*Dunaliella salina*	Chlorophyta, Chlorophyceae	0.827
*Thalassiosira pseudonana*	Stramenopiles, Bacillariophyta	0.825
*Heterosigma akashiwo*	Stramenopiles, Raphidophyceae	0.816
*Asterionella formosa*	Stramenopiles, Bacillariophyta	0.816
*Lithodesmium undulatum*	Stramenopiles, Pelagophyceae	0.812
*Aureococcus anophagefferens*	Stramenopiles, Bacillariophyta	0.812
*Didymosphenia geminata*	Stramenopiles, Bacillariophyta	0.807
**Lowest 15**		
*Euglena gracilis*	Euglenozoa, Euglenida	0.500
*Nephroselmis olivacea*	Chlorophyta, Prasinophytes	0.476
*Cyanidium caldarium*	Rhodophyta, Bangiophyceae	0.476
*Mesotaenium endlicherianum*	Streptophyta, Zygnemophyceae	0.474
*Galdieria sulphuraria*	Rhodophyta, Bangiophyceae	0.461
*Alveolata sp*.	Alveolata, Chromerida	0.448
*Nicotiana tabacum*	Streptophyta, Embryophyta	0.430
*Oryza sativa*	Streptophyta, Embryophyta	0.416
*Chromera velia*	Alveolata, Chromerida	0.415
*Klebsormidium flaccidum*	Streptophyta, Klebsormidiophyceae	0.400
*Theileria parva*	Alveolata, Apicomplexa	0.391
*Helicosporidium sp*.	Chlorophyta, Trebouxiophyceae	0.388
*Trebouxiophyceae sp*.	Chlorophyta, Trebouxiophyceae	0.377
*Pinus thunbergii*	Streptophyta, Embryophyta	0.366
*Cryptomonas paramecium*	Cryptophyta, Cryptomonadales	0.364

#### A combined measurement of selection

**Fig 3:**
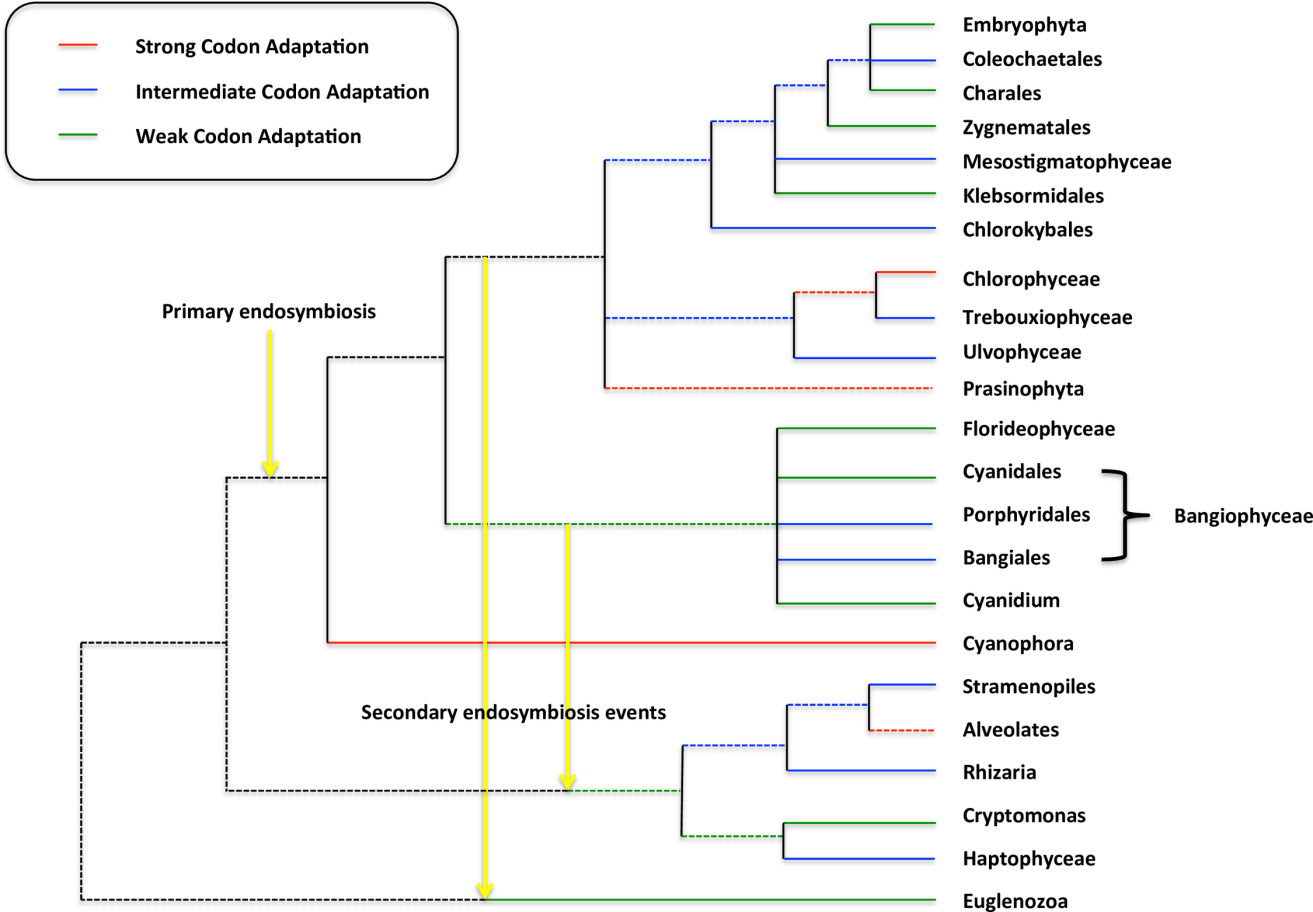
Strength of codon adaptation across lineages. A phylogeny of plastids with the strength of codon adaptation indicated for different lineages. Strength of selection is based on the S_pca_ measure described in the text and given in S1 Table. An average value for the plastids within a given lineage greater than 1 is considered strong adaptation and an average value less than -1 is considered weak adaptation. A dashed line indicates variation within the genomes of that lineage. The primary endosymbiont is indicated as are the two proposed secondary events, one from green plant ancestors to the Euglenoids and another from the red algae ancestors to the lineage leading to extant Cryptophytes, Alveolates, Stramenopiles and Haptophytes (see text). Branches preceding the endosymbiosis are shaded black and indicate a lack of a plastid. The phylogeny overall is based on the general relationships from different sources [13,14].

The three analyses presented above, the resampling test, the S values and the maximal CAI values, measure different aspects of codon adaptation. In an attempt to unify them we followed the example of [26] and generated a single summary statistic of codon adaptation by performing a principal component analysis on the percent rejection in the resampling test, S_3_ values and maximal CAI and taking the value along the first principal component for each genome. This summary statistic, which we call S_pca_, is given in S1 Table for all genomes with a valid S_3_. (S_3_ was chosen over S since more genomes had a valid S_3_ measurement.) The results are summarized in Table 7 and presented graphically in Fig 3, which shows the general strength of codon adaptation in the major plastid lineages.

**Table 7:** Genomes with the strongest and weakest overall codon adaptation as measured by S_pca_.

Genome	Classification	S_pca_
**Highest 15**		
*Oltmannsiellopsis viridis*	Chlorophyta, Oltmannsiellopsis	3.38
*Chlamydomonas reinhardtii*	Chlorophyta, Chlorophyceae	3.24
*Gonium pectorale*	Chlorophyta, Chlorophyceae	3.19
*Dunaliella salina*	Chlorophyta, Chlorophyceae	2.85
*Pedinomonas minor*	Chlorophyta, Pedinophyceae,	2.36
*Kryptoperidinium foliaceum*	Alveolata, Dinophyceae,	2.20
*Asterionellopsis glacialis*	Stramenopiles, Bacillariophyta,	2.18
*Scenedesmus obliquus*	Chlorophyta, Chlorophyceae	2.09
*Monomastix sp*.	Chlorophyta, Prasinophytes,	1.99
*Chlorella sorokiniana*	Chlorophyta, Trebouxiophyceae	1.98
*Pleodorina starrii*	Chlorophyta, Chlorophyceae	1.89
*Schizomeris leibleinii*	Chlorophyta, Chlorophyceae	1.60
*Leptocylindrus danicus*	Stramenopiles, Bacillariophyta	1.57
*Aureococcus anophagefferens*	Stramenopiles, Pelagophyceae	1.52
*Thalassiosira pseudonana*	Stramenopiles, Bacillariophyta	1.46
**Lowest 15**		
*Grateloupia taiwanensis*	Rhodophyta, Florideophyceae	-1.63
*Gracilaria tenuistipitata*	Rhodophyta, Florideophyceae	-1.81
*Euglenaformis proxima*	Euglenozoa, Euglenida	-1.83
*Gracilaria salicornia*	Rhodophyta, Florideophyceae	-2.05
*Mesotaenium endlicherianum*	Streptophyta, Zygnemophyceae	-2.06
*Chara vulgaris*	Streptophyta, Charophyceae	-2.11
*Euglena gracilis*	Euglenozoa, Euglenida	-2.21
*Nicotiana tabacum*	Streptophyta, Embryophyta	-2.23
*Alveolata sp*.	Alveolata, Chromerida	-2.33
*Cyanidium caldarium*	Rhodophyta, Bangiophyceae	-2.39
*Chromera velia*	Alveolata, Chromerida	-2.64
*Oryza sativa*	Streptophyta, Embryophyta	-2.76
*Galdieria sulphuraria*	Rhodophyta, Bangiophyceae	-2.81
*Pinus thunbergii*	Streptophyta, Embryophyta	-2.86
*Cryptomonas paramecium*	Cryptophyta, Cryptomonadales	-4.19

### Correspondence analysis

**Fig 4:**
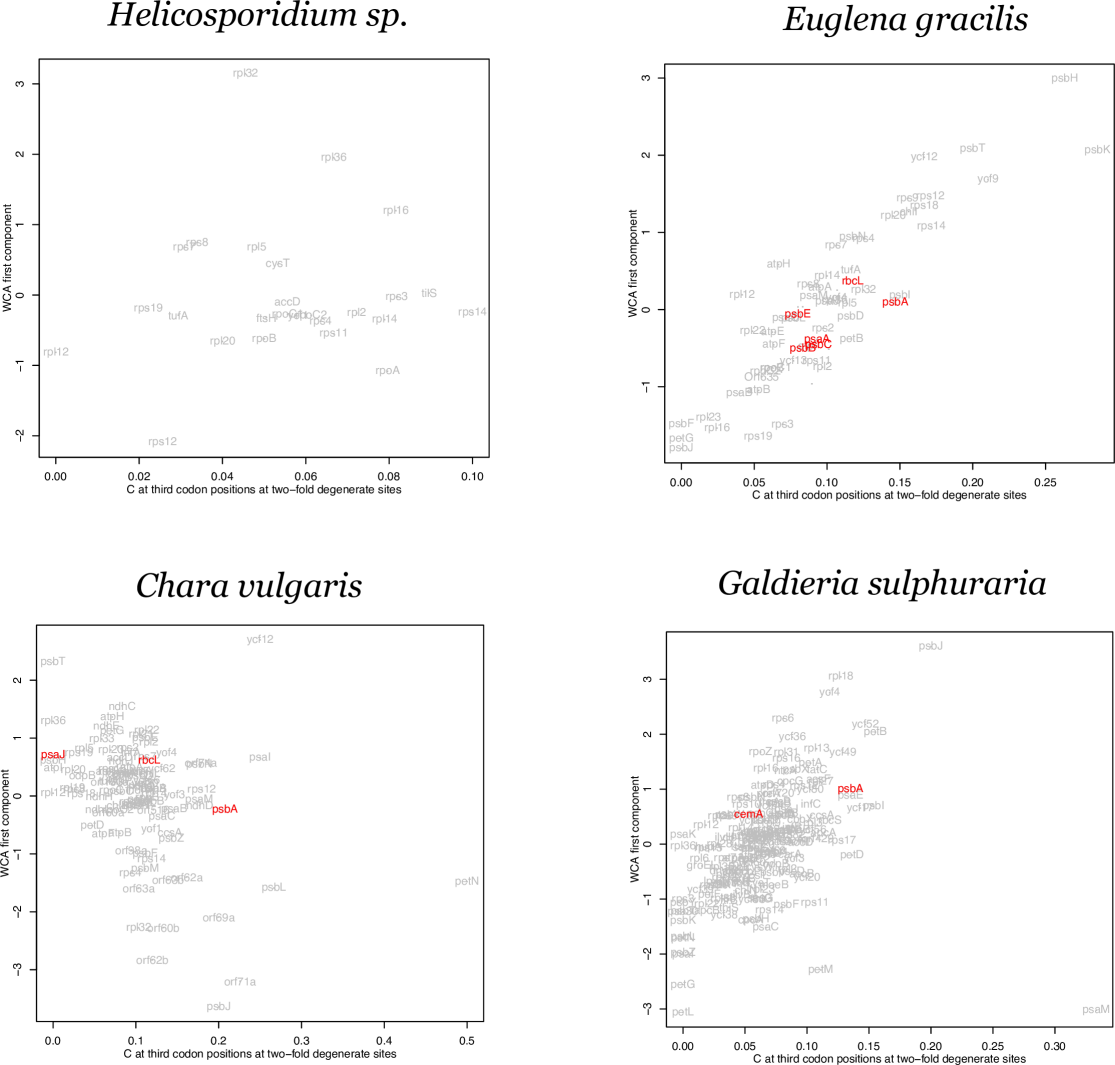
Within-group correspondence analysis (WCA) of codon usage for genomes with low S values. WCA first component plot against %C at two-fold degenerate sites for four plastid genomes inferred to be under weak selection; *Helicosporidium sp*, *Euglena gracilis*, *Chara vulgaris* and *Galdieria sulphuraria*. Genes rejected in the resampling test are highlighted in red. Gene names are given based on NCBI annotation. Full taxon names from the NCBI annotation are given.

**Fig 5:**
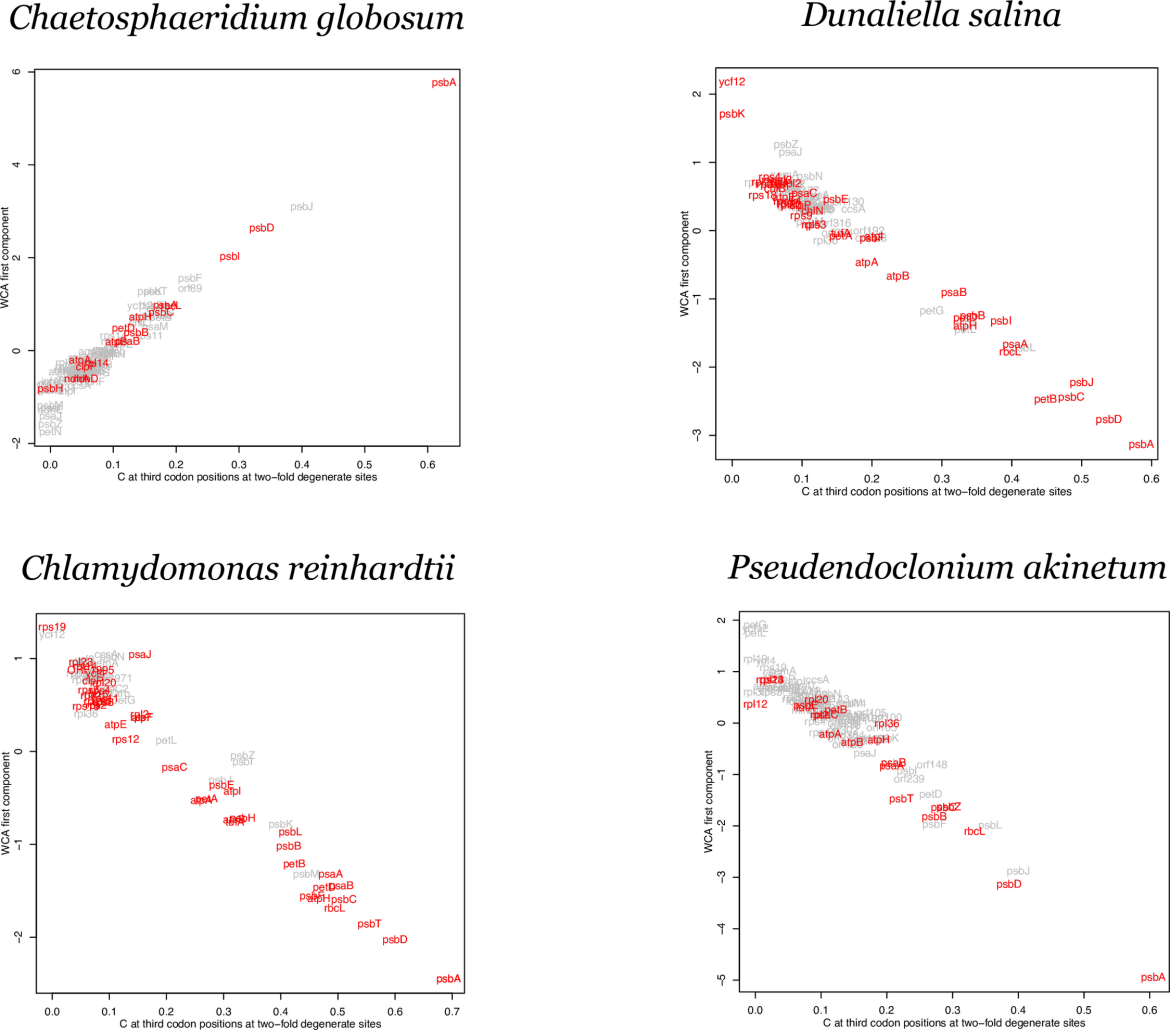
Within-group correspondence analysis (WCA) of codon usage for genomes with high S values. WCA first component plot against %C at two-fold degenerate sites for four plastid genomes inferred to be under strong selection; *Chaetosphaeridium globosum*, *Dunaliella salina*, *Chlamydomonas reinhardtii* and *Pseudendoclonium akinetum*. Genes rejected in the resampling test are highlighted in red. Gene names are given based on NCBI annotation. Full taxon names from the NCBI annotation are given.

Multivariate ordination analysis methods, e.g. principal components analysis and correspondence analysis, have been used to identify orthogonal axes that successively explain the most variation in codon usage among genes. We performed a correspondence analysis to compare to the codon adaptation results. Within-group Correspondence Analysis (WCA) can separate different directions of synonymous codon usage biases (as orthogonal axes) such as those towards NNA and NNT codons, or towards adaptive/favored codons [27,28]. Based on the adaptive pattern observed in the *psbA* genes in Table 1, we compared the first axis of a WCA to the C content at third codon positions over the nine two-fold degenerate amino acids. The results are shown for representative genomes with a low S value or a low rejection rate in the resampling test (Fig 4) and with high S values (Fig 5).

Overall, variation in NNC content is correlated with the primary axis in most genomes (data not shown) with the interesting exceptions being *Theilaria* and *Helicosporidium*, the highly reduced genomes discussed above for which no gene was rejected in our resampling test.

### Genome structure

**Fig 6:**
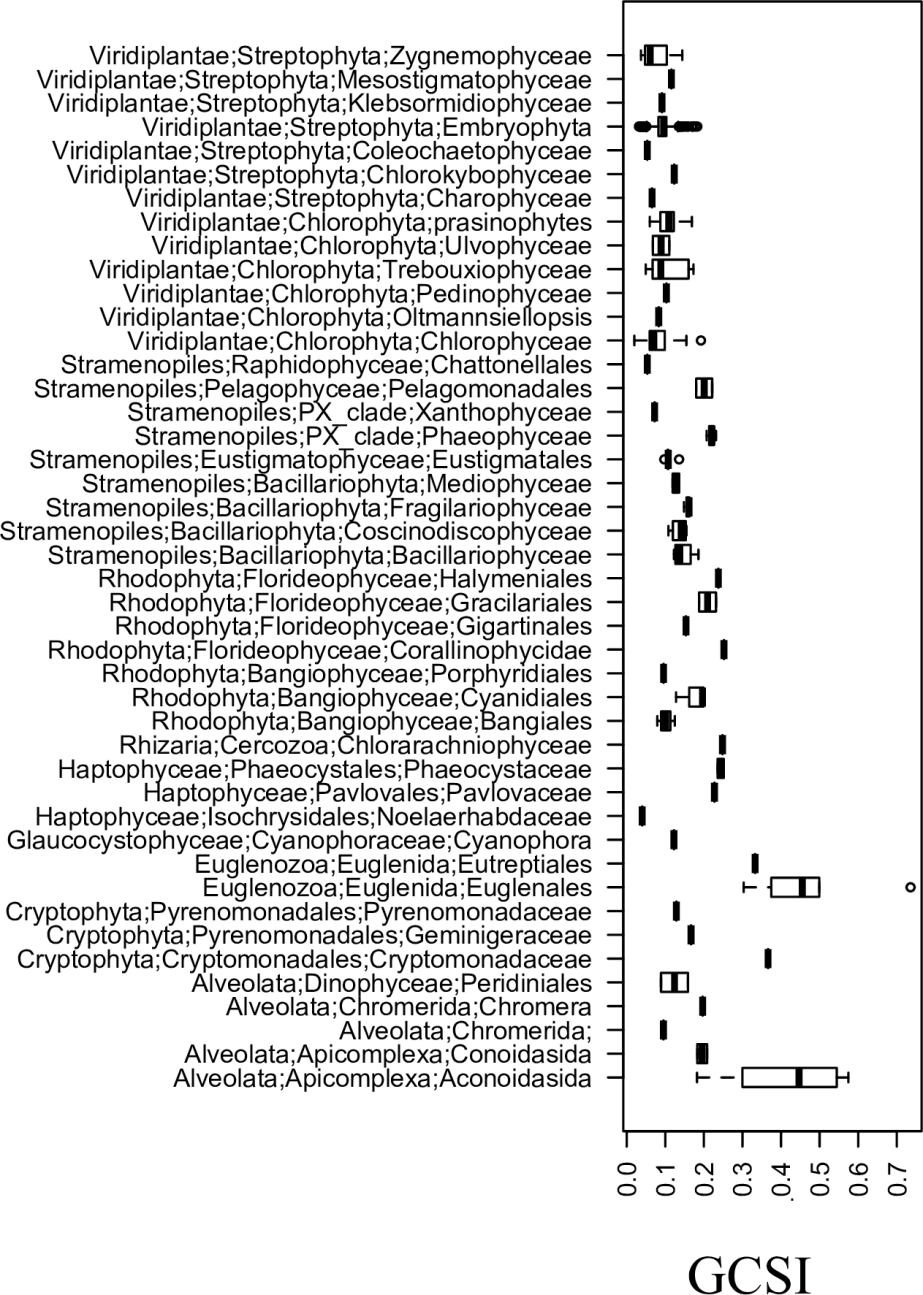
GC Skew in plastid genomes. Box-and-whisker plots summarizing the GC skew index (GCSI) for plastid genomes of different lineages.

The GC skew index (GCSI) value ranges for different plastid genomes are summarized in Fig 6 and all values are presented in S1 Table. Seed plants (represented by *Pinus thunbergii*, *Nicotiana tabacum* and *Oryza sativa* in this study) are not included in the summary since their chloroplast genomes do not replicate from a single origin [42,43].

**Fig 7:**
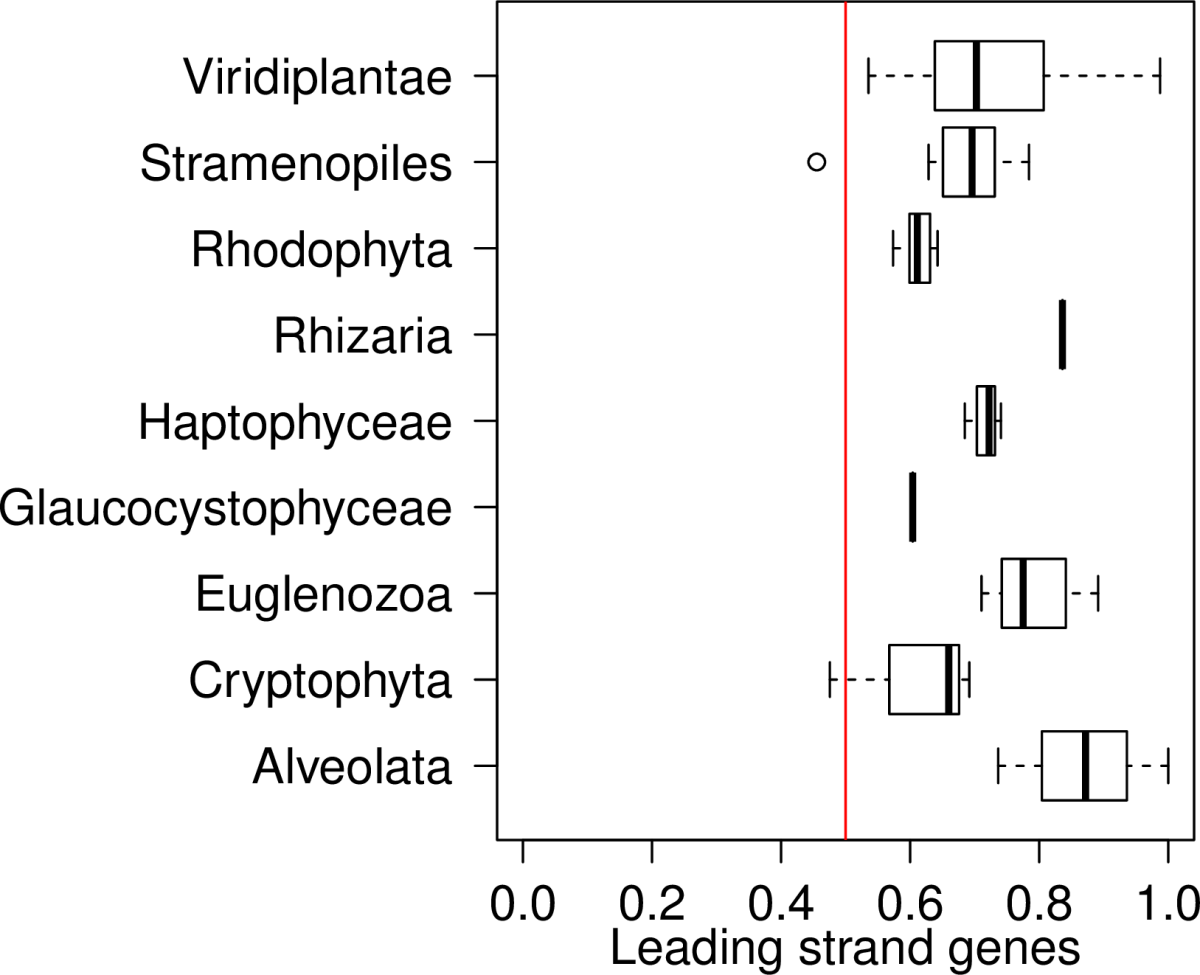
Gene distribution by strand in plastid genomes. Box-and-whisker plots summarizing the distributions of the ratio of leading strand genes to the total number of genes for chloroplasts from nine phyla. A deviation from the ratio = 0.5 (red line) indicates that genes tend to be unevenly distributed between the leading and lagging strands of DNA replication.

We also tested whether or not genes tend to be coded on the leading strand. For the 68 plastid genomes with a GCSI over 0.1 the ratio of inferred leading strand genes to inferred lagging strand genes ranged from 0.46 to 1.00 with a median value of 0.70 (Fig 7). However, there was no indication that genes with high CAI values were coded on the leading strand (data not shown).

## Discussion

Similar to what is observed in bacteria [44] low expression plastid genes display a codon usage with a general bias towards NNA and NNT codons, consistent with the general substitution bias towards A+T that is prevalent across plastid genomes [15,19]. In contrast, highly translated plastid genes, exemplified by *psbA*, across a wide range of taxa display a codon usage pattern that is distinct from low expression genes (Table 1, Figs 1 & 2). Since the bias towards C at the third codon position of some codon groups but towards T, away from both C and A, in other codon groups (Table 1), is not consistent with a general mutational model, some form of selection is required to explain the codon usage of these high expression genes such as *psbA*. We argue that this selection is for translation efficiency: the protein product of *psbA* undergoes oxidative damage during the light reaction and, as a result, is turned over at an extremely high rate making it the major translation product in chloroplasts [25,45]. This makes it likely that the predominant selective pressure generating this codon usage is to increase translation efficiency [15,25] and so we propose that the pattern of codon usage observed in the *psbA* genes in Table 1 and in the other high expression genes reflects codon adaptation in plastids.

This proposal concerning codon adaptation is also consistent with the match between the high frequency of NNC codons and the plastid tRNA population; each of the 38 plastid genomes listed in the tRNA database [24] (http://trna.ie.niigata-u.ac.jp/, see Table 1) has a single tRNA to translate the NNY codon group and in each case the anticodon is complementary to the NNC codon. Since there is no evidence for import of tRNAs from the cytoplasm [46] the tRNA population indicated in Table 1 represents those available for translation of plastid-encoded genes. This codon bias in highly expressed plastid genes is consistent with the proposal that codon selection would be expected to favor C over T in the TTY, TAY, ATY and AAY degenerate groups in all species [7]. The clustering of high translation genes from different taxa suggests that codon adaptation favors the same, or a very similar, set of adaptive codons across plastid genomes. Differences between *psbA*, *rbcL* and *psbC* in Fig 2 would then reflect different levels of codon adaptation based on different levels of translation.

Although a recent article [47] has claimed that plastid genes do not display codon adaptation, this analysis, and some earlier studies [15,25], have shown compelling evidence for codon adaptation in highly expressed plastid genes. The existence of codon adaptation, along with the context-dependent nature of substitutions in the chloroplast genome [16–18], makes analyses of codon usage in plastid genomes more complex than is frequently considered. For example, a number of recent studies, particularly in flowering plants [48–51], failed to account for codon adaptation and context-dependency with the result that their conclusions concerning selection are likely to be incorrect or only partially correct. The conclusions in these analyses concerning selection based on plots of Effective Number of Codon plotted against %G+C content at third codon positions (ENC-GC_3_ plot) were all based on the assumption that every synonymous site should display a uniform equilibrium A+T content. However, because of the context-dependent nature of substitutions [16–18] the expected equilibrium A+T composition varies significantly across sites and this draws into question any conclusions in these studies about selection on codon usage. In another example, Guisinger *et al*. [52] analyzed chloroplast genomes from the family Geraniaceae using an ENC-GC_3_ plot and suggest that relaxed selection and/or mutational biases lead to increased G+C content, which in turn led to a change in codon usage bias. However, since this analysis fails to account for the context-dependency of equilibrium base frequencies the suggestion is unwarranted. Further, given the evidence presented here, analyses of plastid codon usage need to be performed within the context of codon adaptation on highly expressed or translated genes.

The evidence that different plastid lineages show the same adaptive codon usage pattern allows us to measure the degree of ‘fit’ of any gene to this pattern. Although this does not require that the underlying explanation is necessarily codon adaptation, we interpret the degree of fit as a measure of the level of codon adaptation, which would also represent the strength of selection, on a given gene. Therefore, comparisons of genes across genomes will give evidence for different levels of selection in different lineages.

No single measure captures all aspects of codon adaptation so three different metrics of selection strength were presented along with a proposed unifying measurement that we called S_pca_. The resampling test can measure the “breadth” of selection in terms of how many genes may be under selective pressure, but S and maximal CAI provide evidence for the strength of selection on the most highly expressed genes. One drawback to our measurement of S is the low number of genes in the plastid genome, which limits the number of reference genes that can be used. This results in many genomes having no valid S value, something that also limits calculations of S_pca_.

Despite these drawbacks, the data indicate that the Embryophyta and the Rhodophyta other than the Bangiales have relatively weak codon adaptation. In contrast, the evidence suggests that the Chlorophyceae clade of the Chlorophyta, *Cyanophora paradox* (the lone Glaucocystophyceae), the Dinophyceae (Alveolata) and the Bacillariophyta (Stramenopiles) are under relatively strong selection for codon adaptation. Noticeably, *Helicosporidium* sp., *Theileria parva*, which seem to be under very weak codon adaptation as discussed above, did not have valid S_pca_ measures (and so are not given in Table 7). However, given the results of the individual metrics we would infer that these two genomes have among the weakest codon adaptation levels. In fact, given the data above we infer that there is virtually no codon adaptation in either of these two genomes. The mapping in Fig 3 indicates that strong codon adaptation is distributed widely making it difficult to infer whether or not it is an ancestral state but given the presence of at least weak codon adaptation in essentially every plastid genome it is likely that the primary endosymbiont had some degree of adaptation.

Variation across taxa in the level of codon adaptation could have a number of causes [3]. Although the plastid genomes share a relatively conserved set of genes it is likely that the translation level of a specific gene varies across genomes. The results strongly suggest that some genes, *psbA* in particular but also *rbcL* and a few others (Table 2), are highly translated across most, or all, taxa but other genes might have stronger variation. A broad analysis of translation rates across different genes and plastid lineages would be required to assess this, and to compare codon adaptation to translation level generally. This is not a trivial comparison. We have focused on translation level with respect to codon adaptation since selection on codon usage is most likely to act at the level of protein translation. However, it is not established that this is the only, or even predominant, aspect of gene expression that could influence codon adaptation and even the manner in which translation level might be related to codon usage could be extremely complex [3]. Considering this, and the observation that chloroplast protein levels are not significantly influenced by changes in transcript abundance [53], transcript abundance is probably not well correlated with codon adaptation [3]. Therefore, even though some transcript data are available for chloroplasts [54] they are not informative for this type of study. Overall, how the levels of codon adaptation we measure are related to gene expression will require a complex analysis of different stages of expression.

Another consideration is variation in effective population size (N_e_). Species with lower N_e_ values, which would presumably include the seed plants, may have selective pressures that are equivalent to other species, such as green algae, but which are insufficient to overcome genetic drift. Since selection on codon bias involves very small selective differences between synonymous codons [55] we would only expect to find evidence for codon adaptation in species with fairly large effective population sizes. This would be similar to studies of bacteria showing that relatively recent reductions in effective population size have led to a relaxation of selective pressure on codon usage [2,7]. Selective pressures on seed plants may not be different than what exists in the Chlorophyta but a difference in N_e_ would lead to the observed difference in levels of codon adaptation. Overall, the variation in codon adaptation that we observe is likely to result from a combination of all of these factors.

The evidence from our WCA is consistent with the other analyses of codon adaptation. There is a consistent separation of *psbA* along the two axes except for certain genomes that show evidence for relatively weak selection such as *Cyanidium caldarium*, *Euglena gracilis*, *Chara vulgaris*, *Gracilaria salicornia*, *Euglenaformis proxima*, and *Galdieria sulphuraria*. Interestingly, the genes *psbJ* and *petL* stand out along the primary axis in several genomes. Both of these genes are very short (amino acid length ranges from 39 to 50 for *psbJ*, and from 27 to 62 for *petL*) and so, although it is possible that they are under relatively strong codon adaptation in some genomes, codon sampling error (due to the short gene length) makes it difficult to be conclusive.

In each of the WCA plots (Fig 4 and Fig 5) the genes rejected by the resample study in each genome are highlighted. These genes do no generally display a complete separation from the other genes along the primary axis. This supports the data from Table 1 and Fig 2 that indicated that codon adaptation must involve more than just the NNC codons of two-fold degenerate groups. As stated above, the NNC codons are useful as an estimate of codon adaptation but they are not a complete description. The codon usage of different clusters in Fig 1 show that the usage of NNT codons in four-fold degenerate groups is correlated with the bias towards NNC codons of two-fold degenerate groups in high expression genes (Fig 2). Therefore, although the NNC codons are not a complete measure of codon adaptation, the WCA results strongly support the general proposal concerning codon adaptation across plastid genes.

We also compared codon adaptation to aspect of genome organization or structure. No relationship is observed between our measures of the strength of selection on codon usage, covered above, and degree of skew: GCSI is not correlated with percentage rejection in the resampling, with S or with maximum CAI (data not shown). The lineages with the strongest skews in Fig 6 are the Euglenazoa (such as *E*. *gracilis*), which have weak codon adaptation, and the Alveolata. The Chlorophyta, which have very strong adaptation, show very little skew, lower than the Rhodophyta, which are generally under weak adaptation. The one group of Rhodophyta with strong adaptation, the Bangiales, has relatively low skew. Overall there is no discernible relationship between skew and codon adaptation. Many prokaryotic genomes display asymmetric base composition between the leading and lagging strands of replication [56–59] and some have a tendency to code highly expressed genes on the leading strand [59–61]. Strand asymmetry, or skew, in composition and/or gene content is also observed in some plastid genomes [41,62,63] including *E*. *gracilis* [64] although no systematic analysis of genome skew across different plastids has been performed. The evidence here that plastid genes with high CAI values are not preferentially coded on the leading strand could be due to the fact that high expression genes are under strong selection for codon usage because of pressures at the translation level, not the transcription level.

## Conclusions

Based on the data we propose that a single, general, model can explain the vast majority of the variation in codon usage across plastid genomes. Codon usage is determined by a substitution bias towards A+T and selection for adaptive codons that are favored by selection for translation efficiency, as with the basic model presented for bacteria [7]. Given the similarity in the codon usage of the *psbA* gene from different plastid genomes, the evidence indicates that although there is variation in the strength of selection across genomes and genes, the set of adaptive codons is the same across all plastid genomes, possibly as a result of similar tRNA gene contents (see S4 Table).

## Supporting Information

S1 Table Taxonomic information and summary statistics for the 103 genomes analyzed in this study. (XLSX)

S2 Table Codon fitness values, as defined in the Materials and Methods, used to calculate CAI values. (XLSX)

S3 Table Cumulative codon usages for the high and low expression genes clustered in Fig 1. (DOCX)

S4 Table tRNA genes coded in the curated 38 plastid genomes as indicated in the Materials and Methods. Presence (1) or absence (0) of a complementary tRNA is indicated. Some genomes contain multiple tRNA genes with the same anticodon sequence but these are not enumerated.
(XLSX)
