# Supplementary material for: Codon Adaptation of Plastid Genes
Source: PLoS One. 2016 May 19;11(5):e0154306. doi: 10.1371/journal.pone.0154306 (PMC4873144; doi:10.1371/journal.pone.0154306)
Supplement: S3 Table — (DOCX) [file pone.0154306.s003.docx]

**Supplemental Table 3: Codon usage in genes from Fig. 2**

| Codon^1^ | tRNA^2^ | All *psbA* | All *rbcL* | All *psbC* | Low Exp. Genes |
| --- | --- | --- | --- | --- | --- |
|  |  |  |  |  |  |
| AGT | 0 | 153 | 95 | 180 | 1091 |
| **AGC** | 38 | **259** | **28** | **47** | **322** |
|  |  |  |  |  |  |
| AAT | 0 | 210 | 259 | 457 | 3062 |
| **AAC** | 38 | **812** | **446** | **263** | **681** |
|  |  |  |  |  |  |
| TAT | 0 | 122 | 411 | 436 | 2042 |
| **TAC** | 38 | **484** | **405** | **248** | **469** |
|  |  |  |  |  |  |
| TTT | 0 | 293 | 355 | 757 | 2300 |
| **TTC** | 38 | **950** | **423** | **429** | **422** |
|  |  |  |  |  |  |
| CAT | 0 | 132 | 196 | 333 | 1022 |
| **CAC** | 38 | **340** | **246** | **216** | **266** |
|  |  |  |  |  |  |
| ATT | 0 | 560 | 707 | 770 | 3544 |
| **ATC** | 36 | **706** | **266** | **257** | **580** |
| ATA | 0 | 23 | 39 | 158 | 1580 |
|  |  |  |  |  |  |
| TGT | 0 | 90 | 280 | 112 | 451 |
| **TGC** | 36 | **48** | **31** | **15** | **109** |
|  |  |  |  |  |  |
| GAT | 0 | 239 | 817 | 428 | 2470 |
| **GAC** | 38 | **128** | **332** | **131** | **461** |
|  |  |  |  |  |  |
| ACA | 38 | 210 | 501 | 364 | 1480 |
| ACT | 0 | 35 | 45 | 47 | 344 |
| ACC | 23 | 8 | 35 | 101 | 320 |
| ACG | 0 | 519 | 662 | 410 | 1309 |
|  |  |  |  |  |  |
| CCA | 38 | 348 | 373 | 482 | 1176 |
| CCT | 0 | 361 | 358 | 421 | 1249 |
| CCC | 4 | 0 | 9 | 56 | 266 |
| CCG | 0 | 22 | 27 | 52 | 265 |
|  |  |  |  |  |  |
| GCA | 38 | 535 | 647 | 567 | 1214 |
| GCT | 0 | 1080 | 1080 | 822 | 1465 |
| GCC | 3 | 21 | 47 | 81 | 374 |
| GCG | 0 | 54 | 97 | 82 | 265 |
|  |  |  |  |  |  |
| GGA | 33 | 206 | 228 | 565 | 1558 |
| GGT | 0 | 1225 | 1439 | 1462 | 1853 |
| GGC | 35 | 87 | 94 | 202 | 498 |
| GGG | 0 | 12 | 56 | 97 | 467 |
|  |  |  |  |  |  |
| GTA | 38 | 618 | 690 | 524 | 1351 |
| GTT | 0 | 525 | 574 | 558 | 1873 |
| GTC | 21 | 6 | 22 | 34 | 382 |
| GTG | 0 | 42 | 62 | 99 | 382 |
|  |  |  |  |  |  |
| TCA | 38 | 290 | 215 | 349 | 1228 |
| TCT | 0 | 659 | 427 | 461 | 1355 |
| TCC | 23 | 25 | 27 | 67 | 307 |
| TCG | 7 | 19 | 22 | 53 | 331 |
|  |  |  |  |  |  |
| CTA | 38 | 277 | 213 | 232 | 742 |
| CTT | 0 | 185 | 339 | 295 | 1147 |
| CTC | 11 | 1 | 3 | 37 | 214 |
| CTG | 0 | 13 | 26 | 44 | 221 |
| TTA | 37 | 953 | 973 | 1143 | 4203 |
| TTG | 30 | 52 | 64 | 125 | 769 |
|  |  |  |  |  |  |
| CGA | 0 | 5 | 51 | 66 | 998 |
| CGT | 38 | 528 | 797 | 418 | 1315 |
| CGC | 0 | 73 | 54 | 37 | 332 |
| CGG | 23 | 0 | 16 | 12 | 190 |
| AGA | 38 | 53 | 196 | 205 | 1369 |
| AGG | 4 | 0 | 14 | 53 | 251 |
|  |  |  |  |  |  |
| GAA | 37 | 655 | 962 | 703 | 3176 |
| GAG | 0 | 209 | 185 | 148 | 703 |
|  |  |  |  |  |  |
| AAA | 37 | 39 | 769 | 394 | 4675 |
| AAG | 0 | 16 | 162 | 96 | 763 |
|  |  |  |  |  |  |
| CAA | 38 | 318 | 474 | 279 | 2679 |
| CAG | 0 | 18 | 65 | 48 | 543 |

1 – Cumulative codon usages are given for the *psbA, rbcL* and *psbC* genes and for the low expression genes in Fig. 2. The NNC codons of the two-fold degenerate groups are in bold. The AGT and AGC codons of Serine are grouped with the NNY two-fold degenerate codon groups separate from the TCN Serine codons.

2 – Number of the 38 plastid genomes in the tRNA database at http://trna.ie.niigata-u.ac.jp/ that have a tRNA complementary to the codon.
